# Supplementary material for: NOTCH Signaling in Mantle Cell Lymphoma: Biological and Clinical Implications
Source: Int J Mol Sci. 2023 Jun 17;24(12):10280. doi: 10.3390/ijms241210280 (PMC10299455; doi:10.3390/ijms241210280)
Supplement: Supplementary file 1 [file ijms-24-10280-s001.zip › ijms-2428883-supplementary.pdf]

Table S1.

Mantle cell lymphoma[1]		T-cell acute lymphoblastic leukemia[2]	
**Upregulated**	**Downregulated**	**Upregulated**	**Downregulated**
ABCF2	ADAM28	ABCA1	ABTB2
AFMID	AKAP17A	ABCB9	ADAMTS1
AIMP2	ATP6V0A1	ACADL	ADAP1
ALKBH2	BACH2	ACKR3	ADGRG1
AMD1	BCL2	ADTRP	AMPH
BRIX1	CCM2	AIPL1	ANGPT4
BYSL	CD180	ALLC	ANGPTL7
BZW2	CHD2	AMOTL2	ANKH
C17orf96	CIC	AOPEP	ANXA2P3
C1QBP	CKAP4	ARHGAP6	ASB9
CCDC86	CLCN6	ARSD	ATP1B2
CCT2	CNN2	ATP8B3	ATP8A1
CCT5	CYTH1	BAIAP2	BARX2
CCT6A	CYTH2	BBOX1	BBS10
CD44	DDAH2	BCAS1	BTG2
CD48	DDB2	C3AR1	C6orf15
CEBPZ	DERL3	C3orf18	C8B
CEPT1	DGKD	CA2	CA5B
CIRH1A	DOPEY2	CA4	CALB1
CRYZ	DPYSL2	CA8	CASKIN2
CSTF3	E2F2	CAPN10	CAVIN2
CTPS1	ESYT1	CCDC70	CCNA1
CYCS	FADS1	CCDC81	CCNP
DARS	FADS3	CCNK	CDC37L1
DCAF13	FGD4	CD209	CDHR1
DCTPP1	FNDC3A	CD27	CDHR2
DDX18	HDAC5	CD82	CDKAL1
DDX21	HDAC7	CEL	CDKL1
DHX33	INPPL1	CHIA	CDKN1A
DIMT1	IQGAP3	CHRND	CHRNA4
DKC1	KIAA0430	CHST1	CHST8
DNPH1	KIF3C	CIDEB	CIB2
DPH2	KLF6	CLIC2	CLDN16
EBNA1BP2	LAPTM5	CLSTN3	CLMN
EFHD2	LDLRAP1	CNGB3	COL4A1
EIF2S1	LRMP	COQ8B	CPA2
EIF3J	MEF2B	CPNE6	CPB1
EIF4E	MIR600HG	CR2	CPEB3
ELFN1	MMP11	CRH	CRIP1
ETF1	MTHFR	CRHR2	CRYBG2
FABP5	MVD	CRMP1	CRYGC
FAM208B	MYO18A	CST1	CRYM
FARSB	NEAT1	CXCR3	CTDP1

FASTKD2
FJX1
FKBP4
GCNT1
GDI2
GEMIN6
GFM1
GNL2
GNL3
GNPNAT1
GRPEL1
GRWD1
GTPBP4
HEATR3
HK2
HNRNPAB
HSP90AB1
HSPA9
HSPD1
HSPE1
IDH3A
IKBIP
IRF2BP2
KIAA0226
LAMP3
LARP4
LRRC58
LYAR
M6PR
MARS2
MAT2A
MCCC2
METTL1
MRPL12
MRPL3
MRPL36
MRPS35
MRTO4
MYBBP1A
MYC
NAA15
NAA50
NAT10
NDUFAF4
NIP7
NME1
NOB1
NOLC1
OAS1
PBXIP1
PHACTR1
PLP2
PRDM1
PRDM2
RASGRP3
SEMA4A
SETX
SLC23A2
SLC9A7
SLCO4C1
ST3GAL1
STAT2
TNRC6B
TRANK1
VIM

CYP4F2
CYP7B1
DCLK2
DDX4
DIO2
DLK1
DNAJC3
DOC2A
DRAM1
E2F2
EPB41L4A
FAIM
FAM30A
FAT2
FCGRT
FCN1
FMNL2
FSTL3
FUT3
GABRG3
GGTLC1
GNAT3
GPR15
GPR32
GPR65
GPR68
GUCA2A
GUCY1A1
GZMB
HCRT
HES1
HLA-DRA
HOXA3
HS1BP3
ICOS
ID1
ID4
IFI30
IGLC2
IL11
IL26
IL7R
INSM1
IPO8
ITGA7
ITGA9
KCNA1
KCNA5
CTF1
DAAM2
DCLRE1A
DGKE
DGKI
DHX32
DLGAP1
DPT
EFNA3
EPN3
ESM1
ETNPPL
FAM215A
FARP2
FGF13
FGF16
FKBP9
FLT3
FOXP3
FYB1
GABRR1
GDNF
GNG11
GPRASP3
GRIN1
GSN-AS1
GSTA3
GTF3C5
GUCA1B
HABP4
HHLA2
HMOX1
HPCAL4
HSPA1A
HTN1
IFIT1
INAVA
INPP1
IQCC
ITPKC
KCND1
KCNH2
KCNK12
KCNN4
KIF13B
KLHL21
KLHL26
KLRD1

NOP16
NOP2
NUDCD1
NUS1
ODC1
PA2G4
PAICS
PAK1IP1
PCBP3
PDCD5
PHF5A
PINX1
PNO1
PNP
POLR1B
POLR2D
PPA1
PPARGC1B
PPIF
PRMT3
PSME3
PTP4A1
RAD23B
RCL1
RCN1
RIOK1
RPRD1A
RRP1B
RRP9
RRS1
RSL1D1
SEH1L
SERBP1
SERPINB9
SIAH2
SLC25A33
SLC3A2
SNHG16
SRSF7
STEAP1
TFRC
TMA16
TOMM5
TP53RK
TRMT6
TSPAN11
TWISTNB
TYW3

KCNE1
KCNN1
KLF15
KLF2
KLHL12
KRT9
LGALS4
LHX6
LINC01140
LMTK2
LPAR6
LRP4
LY6G6C
LY6G6E
LZTFL1
MAGEC3
MAPK13
MMP25
MRM1
MYCN
MYO1A
MYO1D
MYO1E
MYO7B
NID1
NOTCH3
NSUN6
OGDH
OPRPN
OR2H1
ORAI2
ORM1
PBOV1
PGLYRP1
PHKG1
PKD2L1
PLAT
POF1B
PPARGC1A
PPFIA4
PPIEL
PSPN
PTCRA
RAB3IL1
RAMP2
RASSF8
RCBTB2
RHO

KRT18
KRT8P12
KSR1
LCE2B
LDOC1
LHCGR
LINC01565
LMAN1
LMO3
MAP3K1
MAP3K2
MEFV
MFSD6
MN1
MTG1
MYF5
NAA80
NEU2
NEUROG3
NME5
NPTX1
NPTXR
NPY6R
NR6A1
NYNRIN
OR10H3
P2RX3
P2RY1
P3H3
PAIP2B
PF4V1
PIAS3
PIM2
PKDREJ
PLA2G7
PNPLA3
PPEF2
PPP1R13L
PRKACA
PRKG1
PROC
PRSS50
RAB23
RAG1
RAG2
RANBP17
RASGRP1
RDH5

UCK2
UMPS
UNG
URB2
UTP18
WDR12
WDR3
WDR43
YRDC
YWHAG
ZC3H15
ZFAT

RPL39L
SAMD4B
SCNN1A
SELE
SEMG2
SERPINE1
SH3TC1
SHC2
SHCBP1L
SLC28A3
SLC45A2
SLN
SLPI
SNED1
SP140
SPANXC
SPRR2B
SPRY4
SUSD4
TBC1D29P
TBX3
TFPI2
TM4SF20
TMEM132A
TMEM92
TMPRSS6
TNFRSF8
TNFSF10
TRGV7
TRPC3
TRPV6
TUBB2B
UTF1
VEGFD
VN1R1
VNN2
VPREB3
WIF1
ZBTB16
ZC3H12A
ZFP37
ZNF221
ZNF74
ZSCAN5A

RFPL1
RGS2
RHCE
RNF39
RP2
RPS6KA4
RRAD
RUNX2
S100A8
SCN8A
SCTR
SETD1A
SLC17A9
SLC30A10
SLC39A4
SLC5A1
SLC5A4
SLC6A14
SLC9A7
SPTBN2
SRGAP2
SSH3
STARD8
STAT2
SYT12
TENM1
TFDP3
TGFB1I1
TGFBR1
TLX1
TMEM59L
TMEM74B
TNFSF8
TNNI3
TNS1
TPPP
TTC21B
TUBBP5
UPB1
WDR7
YBX2
ZBBX
ZNF112
ZNF230
ZNF507
ZNF671
ZSCAN31

Table S1: Genes that are up and downregulated by NOTCH signaling in mantle cell lymphoma and in T-cell acute lymphoblastic leukemia. Genes are listed in alphabetical order.

1. Ryan, R. J. H.; Petrovic, J.; Rausch, D. M.; Zhou, Y.; Lareau, C. A.; Kluk, M. J.; Christie, A. L.; Lee, W. Y.; Tarjan, D. R.; Guo, B.; Donohue, L. K. H.; Gillespie, S. M.; Nardi, V.; Hochberg, E. P.; Blacklow, S. C.; Weinstock, D. M.; Faryabi, R. B.; Bernstein, B. E.; Aster, J. C.; Pear, W. S., A B Cell Regulome Links Notch to Downstream Oncogenic Pathways in Small B Cell Lymphomas. *Cell Rep* **2017,** 21, (3), 784-797.
2. Dohda, T.; Maljukova, A.; Liu, L.; Heyman, M.; Grander, D.; Brodin, D.; Sangfelt, O.; Lendahl, U., Notch signaling induces SKP2 expression and promotes reduction of p27Kip1 in T-cell acute lymphoblastic leukemia cell lines. *Exp Cell Res* **2007,** 313, (14), 3141-52.
